# Treatment Algorithm for Chronic Idiopathic Constipation and Constipation-Predominant Irritable Bowel Syndrome Derived from a Canadian National Survey and Needs Assessment on Choices of Therapeutic Agents

**DOI:** 10.1155/2017/8612189

**Published:** 2017-02-08

**Authors:** Yvonne Tse, David Armstrong, Christopher N. Andrews, Alain Bitton, Brian Bressler, John Marshall, Louis W. C. Liu

**Affiliations:** ^1^University of Toronto, Toronto, ON, Canada; ^2^McMaster University, Hamilton, ON, Canada; ^3^University of Calgary, Calgary, AB, Canada; ^4^McGill University, Montreal, QC, Canada; ^5^University of British Columbia, Vancouver, BC, Canada

## Abstract

*Background*. Chronic idiopathic constipation (CIC) and constipation-predominant irritable bowel syndrome (IBS-C) are common functional lower gastrointestinal disorders that impair patients' quality of life. In a national survey, we aimed to evaluate (1) Canadian physician practice patterns in the utilization of therapeutic agents listed in the new ACG and AGA guidelines; (2) physicians satisfaction with these agents for their CIC and IBS-C patients; and (3) the usefulness of these new guidelines in their clinical practice.* Methods*. A 9-item questionnaire was sent to 350 Canadian specialists to evaluate their clinical practice for the management of CIC and IBS-C.* Results*. The response rate to the survey was 16% (*n* = 55). Almost all (96%) respondents followed a standard, stepwise approach for management while they believed that only 24% of referring physicians followed the same approach. Respondents found guanylyl cyclase C (GCC) agonist most satisfying when treating their patients. Among the 69% of respondents who were aware of published guidelines, only 50% found them helpful in prioritizing treatment choices and 69% of respondents indicated that a treatment algorithm, applicable to Canadian practice, would be valuable.* Conclusion*. Based on this needs assessment, a treatment algorithm was developed to provide clinical guidance in the management of IBS-C and CIC in Canada.

## 1. Introduction

Chronic idiopathic constipation (CIC) and irritable bowel syndrome with predominant constipation (IBS-C) are common gastrointestinal disorders in North America, with CIC affecting 15–25% [1] of the general population and IBS-C affecting 11.8% [2]. Canada is amongst one of the countries that carry a high rate of IBS in the world, with a prevalence estimated to be 1 in 7 in the population and an incidence of 120,000 new cases per year [2, 3]. IBS-C is more common in females and younger adults of less than 50 years of age, whereas CIC affects females and older adults more commonly [4]. CIC and IBS-C impair patient's quality of life (QoL), similar to those suffered from asthma and rheumatoid arthritis, and negatively impact socioeconomics of the society [5, 6].

Although the Rome III diagnostic criteria define CIC and IBS-C as separate entities, with IBS-C being distinguished by the presence of abdominal pain and discomfort, these criteria cannot distinguish reliably between CIC and IBS-C. Many patients have features of both disorders and their diagnosis may change over time [7, 8]. In 2014, America College of Gastroenterology (ACG) published a systematic review on the efficacy of available therapy in IBS and CIC [9]. Earlier in 2013, American Gastroenterological Association (AGA) published a medical position statement on constipation [10], followed by an AGA guideline on pharmacological management of IBS in 2014 [11]. However, all these guidelines did not provide direction in terms of the priority of when the therapeutic agents are recommended to be used to optimize management in patients suffering from CIC and IBS-C.

Using a national survey, we aimed to evaluate Canadian specialists' practice patterns in the utilization and satisfaction of various therapeutic agents for their CIC and IBS-C patients. The survey also addressed if the respondents found the new ACG guidelines useful for them to prioritize which agents to use in their clinical practice. Informed by the result of this needs assessment survey, a treatment algorithm was developed for the management of CIC and IBS-C. This treatment algorithm aligned with the recently published literature (ACG and AGA guidelines), while also considering the limitations of these American guidelines in the Canadian practice.

## 2. Methods

A questionnaire was sent to 350 Canadian physicians. Data were collected over a 4-month period in early 2015 (CARE, Toronto, ON) to evaluate respondents' area of practice and their constipation management patterns in patients with CIC and IBS-C.

### 2.1. Questionnaire

The questionnaire (the Appendix) evaluated physicians' perceptions and practices with respect to the management options available in Canada for the treatment of constipation in patients with CIC and IBS-C. Treatment options were divided into stool softeners, fibre supplements, stimulant laxatives, osmotic laxatives, prokinetic agents, GCC agonists, and “other.” Respondents were asked to rank the order in which they would use these treatment options for patients with inadequate fibre intake, slow-transit constipation, functional outlet constipation, and IBS-C and their satisfaction with the outcomes of these treatment agents for their patients with CIC or IBS-C. Satisfaction with treatment response was recorded on a 5-point scale from 1 (very unsatisfied), 2 (dissatisfied), 3 (neutral/not sure), 4 (satisfied), to 5 (very satisfied). Additional questions regarding strategies used by referring physicians, awareness of the most recent ACG guidelines, and questions relating to other practice characteristics were also included.

### 2.2. Analysis of the Practice Survey

In order to statistically determine which treatment options were considered most satisfactory for CIC and IBS-C (the Appendix: Question (6) (A) and (B)), the questions were scaled based on their rating (number of responses for a particular rating divided by the total number of responses) and then grouped based on whether respondents thought they were not satisfied (the Appendix: Question (6) ratings 1 and 2) or satisfied (the Appendix: Question (6) ratings 4 and 5). These *t* values were then calculated to determine the clinical significance of each.

## 3. Results

### 3.1. Practice Preferences and Satisfaction of Therapeutic Agents

Completed questionnaires were returned by 55 of 350 (16%) physicians although efforts had been made to collect response via both hardcopy survey and online survey tool with reminders to encourage participants to respond.  Table 1 showed the demographic distribution of the respondents. Almost all (96%) respondents reported that they followed a standard, graduated, or stepwise approach for managing constipation; however, only 24% of respondents reported that referring physicians used a graduated or stepwise approach in managing patients with constipation. Most respondents reported that patients (59%) and referring physicians (62%) were reluctant to move from over-the-counter (OTC) therapies to prescription medications.

**Figure 1:**
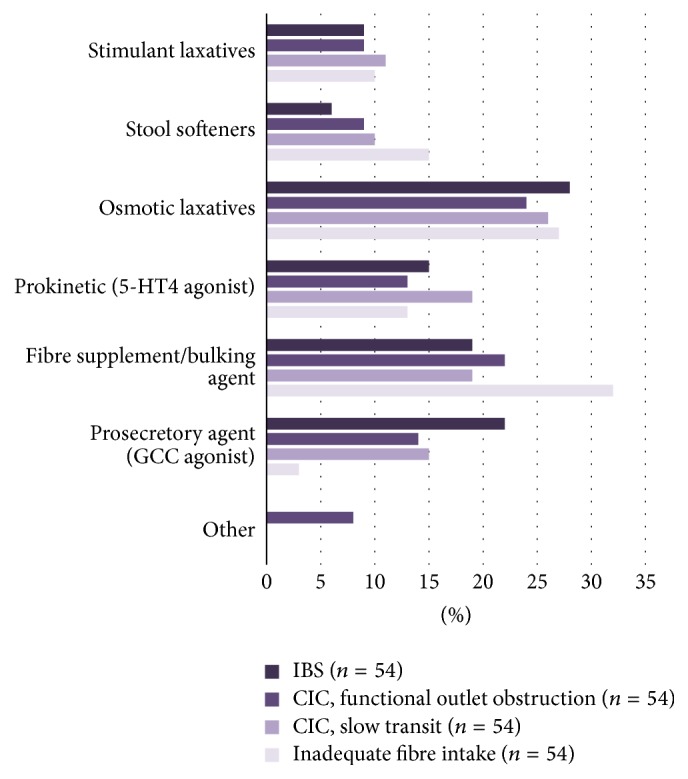
Types of treatment patients would ideally receive based on the type of chronic constipation (the Appendix: Question (4)). Osmotic laxatives were ranked as first-line treatment for both CIC and IBS-C, followed by fibre supplements/bulking agents being second-line treatment for CIC and GCC agonist being second-line treatment for IBS-C.* (CIC*:* chronic idiopathic constipation; IBS-C*:* constipation-predominant irritable bowel syndrome; GCC*:* guanylyl cyclase C)*.

**Figure 2:**
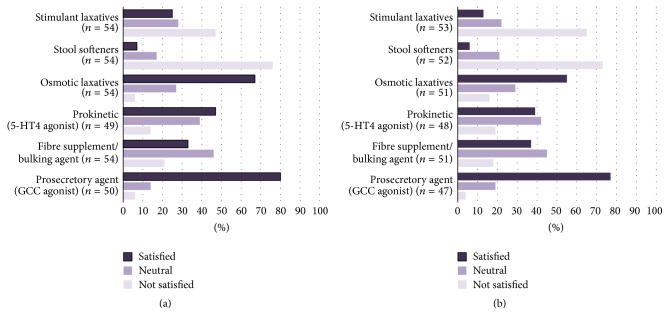
(a) Respondents' satisfaction with treatment options for managing patients with CIC. Stool softeners and stimulant laxatives (*p* = 0.0002) were found to be unsatisfying treatments for CIC compared to other options. On the other hand, GCC agonist (*p* < 0.0001), osmotic laxatives (*p* < 0.0001), 5-HT4 agonist (*p* = 0.0003), and fibre supplements/bulking agents (*p* = 0.0069) were considered to be satisfying treatments for CIC among the treatment options being surveyed.* (CIC*:* chronic idiopathic constipation; GCC*:* guanylyl cyclase C.)* (b) Respondents' satisfaction with treatment options for managing patients with IBS-C. Both stool softeners (*p* < 0.0001) and stimulant laxatives (*p* = 0.038) were unsatisfying treatments for IBS-C compared to other options, whereas GCC agonist (*p* < 0.0001), osmotic laxatives (*p* < 0.0001), 5-HT4 agonist (*p* = 0.003), and fibre supplements/bulking agents (*p* = 0.004) were considered to be satisfying treatments for IBS-C among the treatment options being surveyed.* (IBS-C*:* constipation-predominant irritable bowel syndrome; GCC*:* guanylyl cyclase C)*.

Among the six therapeutic options to manage constipation, the respondents selected osmotic laxatives and fibre supplements/bulking agents as the first-line (ranked 1 or 2 of question (4) in this survey) treatment of both CIC and IBS-C (Figure 1). Stool softeners were shown to be statistically the least satisfying treatment option for patients with CIC (Figure 2(a), *p* = 0.0002) when analyzing the responses from those who rated each treatment option as “1” (question (6) of the survey). When ratings of “1” and “2” (question (6) of the survey) were grouped for analysis, stimulant laxatives were also found to be an unsatisfying treatment option for CIC (*p* = 0.0002). On the other hand, GCC agonist (*p* < 0.0001), osmotic laxatives (*p* < 0.0001), 5-HT4 agonist (*p* = 0.0003), and fibre supplements/bulking agents (*p* = 0.0069) were considered to be satisfying treatments for CIC. For patients with IBS-C, respondents identified both stool softeners and stimulant laxatives as significantly unsatisfying treatment options (Figure 2(b), *p* < 0.0001 and *p* = 0.038, resp.). Similar to the management of CIC, GCC agonist (*p* < 0.0001), osmotic laxatives (*p* < 0.0001), 5-HT4 agonist (*p* = 0.003), and fibre supplements/bulking agents (*p* = 0.004) were considered to be satisfying treatments for IBS-C, with GCC agonist being the most satisfying treatment option (*p* = 0.03 when ratings of “5” were analyzed).

**Figure 3:**
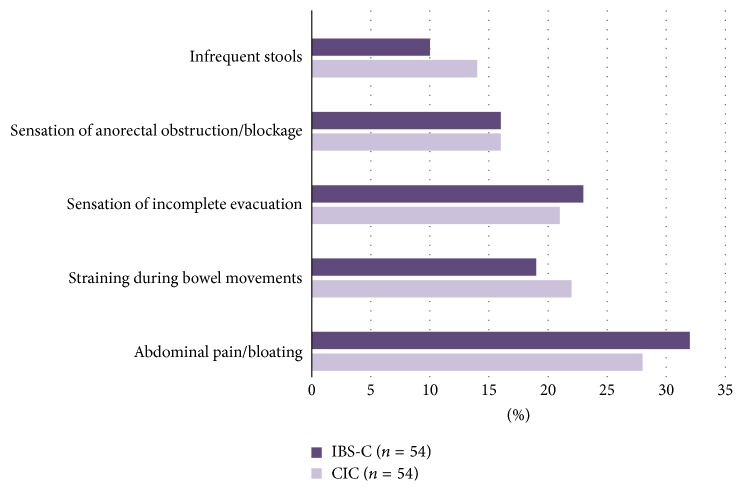
Respondents' perceptions of symptoms that most affect patients' quality of life based on the type of constipation. Abdominal pain/bloating was ranked as being the most burdensome symptom in both CIC (28%) and IBS-C (32%).* (CIC*:* chronic idiopathic constipation; IBS-C*:* constipation-predominant irritable bowel syndrome)*.

**Figure 4:**
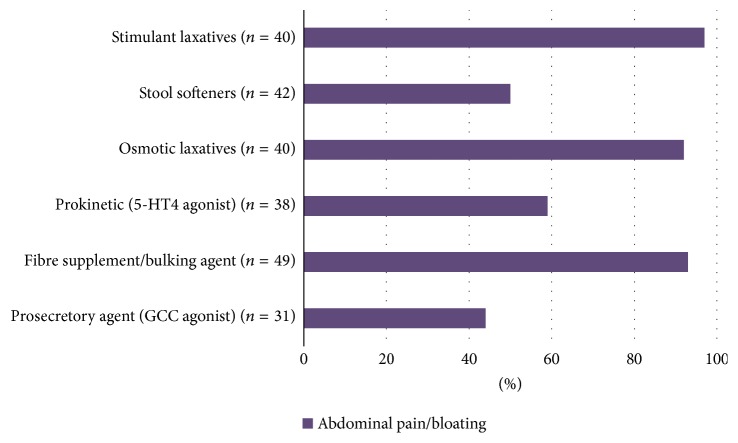
Respondents' perceptions of symptoms that patients most often complain about while being on various therapy options. For abdominal pain/bloating, which was being identified as the symptom that most affects patients' quality of life in both CIC and IBS-C, patients who were prescribed GCC agonist appeared to be least likely to report abdominal pain/bloating, suggesting that this agent may be the most effective to alleviate patients with constipation and abdominal pain/bloating.* (CIC*:* chronic idiopathic constipation; IBS-C*:* constipation-predominant irritable bowel syndrome; GCC*:* guanylyl cyclase C)*.

Abdominal pain/bloating were ranked as being the most burdensome symptoms that affect QoL in patients of CIC and IBS-C (28% and 32%, Figure 3). Our survey indicated that the respondents reported that their patients least likely complained of abdominal pain/bloating while using GCC agonist, suggesting that this agent may be the most effective to alleviate patients with constipation and abdominal pain/bloating and least likely to cause these symptoms as side effects (Figure 4).

### 3.2. Awareness and Perception of the Usefulness of ACG Guidelines

Despite the majority of respondents (69%) being aware of the recent ACG guideline for managing CIC and IBS-C, only half of the respondents (39%, somewhat helpful, 11%, very helpful) found the guideline helpful to prioritize the use of available treatment options. Hence, most of them (69%) expressed great interest in having a treatment algorithm that would be applicable to Canadian practice.

## 4. Discussion

Although efforts were made to increase response rate by using hardcopy survey and online survey tool with reminders being sent to encourage participation, the response rate was only 16% (*n* = 55), which can be a limitation of generalizability of this study. This may reflect the lack of interest and/or knowledge in this field of gastroenterology although this aspect is beyond the scope of this survey. Nevertheless, we believe that the results of this survey still provide useful information on gastroenterologists' practice in the management of IBS-C and CIC in Canada since we speculate that those who responded to this survey were likely to be more familiar with the conditions.

The results of this survey indicate that many specialists believe that referring physicians might not follow a similar stepwise approach to the management of constipation that many specialists use. This may cause unsatisfactory treatment outcomes in some patients, resulting in unnecessary referrals. In addition, results suggest there is the perception that both patients and/or referring physicians may be reluctant to move from OTC therapies to prescription agents such as prucalopride or linaclotide, when the former failed to provide symptomatic relief. This could be due to a number of factors, including ease of access and limited reimbursement for prescription medications, prescriber experience and knowledge of newer treatment options, perceived severity of taking prescription medications, and difference in treatment schedule of OTC versus prescription medications. Responses collected from this survey suggest that there is a need to provide education not only to specialists but also to referring physicians and patients.

Although the Rome diagnostic criteria distinguish IBS-C from CIC, in clinical practice, these conditions are a spectrum of illnesses with overlapping symptoms and interchangeable diagnosis over time in the same patient [7, 8]. This clinical observation is consistent with our survey illustrating that abdominal pain/bloating being most frequently reported by the respondents to be the most burdensome symptoms that affect their patients' QoL in both CIC and IBS-C (Figure 3). The respondents also reported that when GCC agonist was used to treat their patients with constipation, their patients least likely complained of abdominal pain and bloating (Figure 4). We believe that this is the underlying reason the respondents find using GCC agonist the most satisfying in treating their patients with CIC and IBS-C (Figure 2). This survey's outcome was supported by clinical trial observations that GCC agonist, linaclotide, improves both bowel movement frequency and abdominal pain [12–14].

Due to the overall negative impact of CIC and IBS-C on the patients' QoL and the health care system, practice guidelines had been developed. While the recent ACG and AGA guidelines for CIC and IBS-C management are available, there are limitations of applying these guidelines directly in Canadian clinical practice; for example, availability and accessibility of drugs are different between the United States and Canada. In addition, physician adherence to guidelines is another limitation of translating these guidelines in clinical practice. Literature suggests that physician adherence to guidelines is often quite inadequate [15–17]. Adherence can be restricted by several factors, including the length and complexity of guideline content, the presence of multiple guidelines (in different versions or from different groups), and limited access to the guidelines in a clinical practice setting [18].

**Figure 5:**
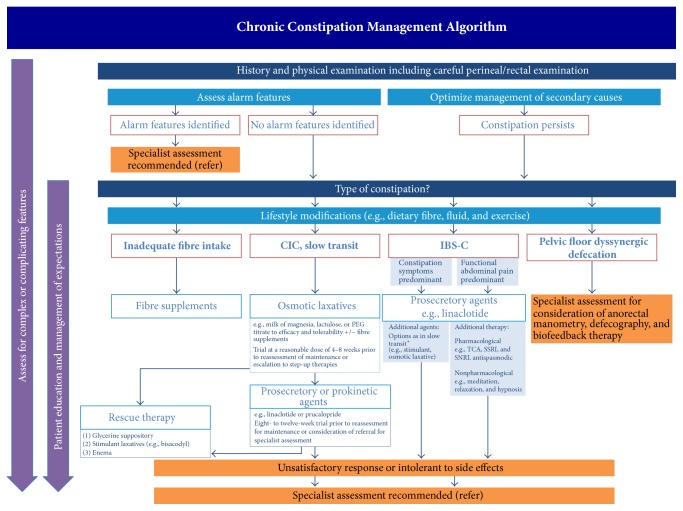
Management algorithm for chronic constipation. The priority usage of each therapeutic option depends on patients' tolerability and affordability. ^*∗*^*There has been no evidence to support the use of prucalopride in patients with IBS-C. (IBS-C*:* constipation-predominant irritable bowel syndrome)*.

Another practical limitation of guidelines, such as the ACG [9] and the AGA [10, 11] guidelines, is that they do not prioritize the use of recommended agents to achieve optimal patient outcomes since head-to-head evaluation of treatment effects of various agents is not usually available. Hence, it will rely on real-world treating physician experience to choose which agent to use when various agents receive strong recommendation. The feedback gathered from the needs assessment survey clearly indicates that a Canadian-centered and evidence- and experience-based algorithm that guides physicians to select the appropriate treatment options is needed. Based on the responses from this survey, available evidences, and experience of the authors, we develop an algorithm to help physicians to manage patients with constipation (Figure 5).

### 4.1. Development of the Treatment Algorithm

#### 4.1.1. History and Physical Examination

A thorough history and physical examination including digital anorectal examination (DARE) are important in identifying secondary causes such as medication-induced constipation (e.g., opioids, calcium-channel blockers, and anticholinergics) and other underlying medical comorbidities (e.g., diabetes, connective tissue diseases, and neurological diseases). If alarming features (such as new onset of symptoms after 50 years of age, rectal bleeding, nocturnal symptoms, significant weight loss, fever, and anemia) or abnormal physical examination are identified, the patient should be referred to a specialist for further assessment to rule out more ominous etiology, such as malignancy.

The purpose of performing a careful DARE is to identify patients who may have dyssynergic defecation (DD) as an etiology of their constipation because management may involve further investigation such as anorectal manometry (ARM) or defecography. However, a careful DARE was found to be accurate with a positive predictive value of over 90% compared to ARM in two independent population cohorts presented with constipation [19, 20]. When DD is identified by DARE in patients presented with constipation, a referral to pelvic health physiotherapists for biofeedback is recommended to optimize treatment outcomes in addition to a regular bowel regimen [21].

A thorough history and physical examination not only allow the physicians to assess alarming features or secondary causes but also allow the physician to establish a therapeutic relationship with the patient. This will provide an opportunity for the physician to address the patient's concerns and to educate the patient about what is being considered to be normal bowel movements, including the natural variation of bowel functions and the range of normal stool frequency, as well as set realistic treatment goals for each individual patient. Dietary and lifestyle modifications (i.e., optimizing dietary fibre and fluid intake and encouraging regular physical activity) should first be considered as initial management. Because of the lack of harmful effect of lifestyle and dietary modification, it is widely accepted and recommended by experts as first-line therapy; despite the lack of strong evidence that these measures improve constipation, they have been shown to improve overall QoL and IBS symptoms severity [22–25].

#### 4.1.2. Treatment Recommendations Based on Subtypes of Constipation

*(1) Inadequate Fibre Intake.* The recommended daily fibre intake is 25–38 g [26]. Fibre supplement helps increase stool weight and improve stool consistency [27] in order to decrease stool transit time [28]. In patients with low fibre intake based on dietary history, it is logical to add fibre supplements. Soluble, such as psyllium, is preferable over insoluble fibre because of the lower propensity to cause associated abdominal symptoms (e.g., bloating or abdominal pain); hence it is better tolerated by most patients [9]. The recommended daily fibre supplement intake is generally up to 12 g per day [9]; the target consumption can be achieved with gradual incremental increases (e.g., 3 g/day increment per week) to avoid side effects such as bloating and flatulence.

*(2) Chronic Idiopathic Constipation and Slow-Transit Constipation*. A thorough history and digital rectal examination along with the Bristol Stool Chart is frequently adequate to diagnose patients with slow-transit constipation. Type 1 or 2 Bristol stool consistency correlates with prolonged colonic transit time [29]. Although sitz marker colonic transit study can be helpful, it is not widely accessible.

In patients with slow-transit constipation, osmotic laxatives are recommended as the first-line pharmacological agents to be used because of their effectiveness, accessibility, and affordability. Milk of magnesia, lactulose, and PEG are the most commonly used osmotic laxatives in Canada. Both lactulose and PEG had randomized-controlled trials (RCTs) to support their use [30–37] while milk of magnesia was based on expert opinions and clinical experience [22, 23]. Milk of magnesia should not be used in patients with renal impairment because of its renal excretion. In general, PEG causes less bloating and flatulence compared to lactulose; thus it may result in better tolerability and compliance by patients.

If patients experience no improvement with their constipation after using osmotic laxatives, GCC agonist (linaclotide) or prokinetic agent (prucalopride) is recommended as the second-line agent for regular use. Rescue therapy (i.e., glycerine suppository, stimulant laxatives, and enema) can be added as adjuvant therapy for occasional use. Suppository and enema are sometimes difficult for patients to administer. There is clinical opinion that stimulant laxatives could cause dependency and cathartic colon if used regularly although direct evidence to support this claim is unavailable.

An adequate trial of osmotic laxative use before stepping up to the second-line agents varies depending on the ability to provide follow-up in clinical practice. We recommend that if a patient does not achieve a satisfactory response by 8 weeks, therapy should be advanced to second-line agents such as prucalopride or linaclotide. Effectiveness of linaclotide and prucalopride in treating constipation has been proven in multiple placebo-controlled RCTs [12–14, 38–40]. The commonly reported side effect of linaclotide is diarrhea. In comparison, prucalopride causes headache, nausea, abdominal pain, and diarrhea in 5–10% treated patients. This side effect profile likely reflects in the result of our survey that the respondents found linaclotide most satisfactory in treating their patients with constipation. Furthermore, in Canada, prucalopride is only approved for use in woman with CIC; linaclotide is approved for adults with CIC.

Stool softeners are not included in our management algorithm because of weak evidence in supporting their use [41, 42]. In fact, a multicenter double-blinded RCT involving 170 patients for two weeks revealed inferiority of sodium docusate compared to psyllium at improving stool frequency [42]. Probiotics are not included in the algorithm currently due to insufficient evidence to support their use [9].

*(3) IBS-C*. A hallmark feature that distinguishes patients diagnosed with IBS-C from CIC is the presence of associated functional abdominal symptoms, namely, abdominal pain and bloating. This has been consistently demonstrated in a previous Canadian population study [6] and in our current survey. Therefore, treatment agents that alleviate both constipation and the associated debilitating functional abdominal symptom would be preferable.

A GCC agonist, linaclotide, has been shown to improve satisfactory complete spontaneous bowel movement frequency and decrease associated abdominal pain [12–14]. Our survey respondents also identified GCC agonist to be the most satisfying in treating their patients with IBS-C. Hence, the authors recommend linaclotide to be used as the first-line pharmacological agent in patients with IBS-C. The dosage of linaclotide indicated for IBS-C is 290 *μ*g daily whereas for CIC is 145 *μ*g daily (Table 2).

Depending on the treatment response, supplemental therapy such as stimulant laxatives and osmotic laxatives can be used in patients requiring further treatment for constipation, or neuropathic agents (selective serotonin reuptake inhibitors, serotonin-norepinephrine reuptake inhibitors, tricyclic antidepressants, or antispasmodics) in patients requiring additional therapy for abdominal pain. It is worth noting that tricyclic antidepressants and antispasmodics often worsen constipation. Nonpharmacological therapy such as meditation, relaxation, or hypnosis has been shown to improve functional abdominal symptoms in patients with IBS [43]. Patients who do not respond or are intolerable to the treatment options outlined in the algorithm are suggested to be referred to specialized centers for further assessment.

## 5. Conclusion

Our Canadian survey suggests that management strategies for CIC and IBS-C among physicians are heterogeneous, which can result in dissatisfaction in treatment response from both patients and physicians. A management algorithm for chronic constipation specifically developed to apply in Canadian practice will help in optimizing treatment outcomes for patients suffering from these functional GI disorders.

## Figures and Tables

**Table 1:** Respondent demographics.

Characteristics	Number (%)
Province:	
(i) East Coast	5 (10%)
(ii) Quebec	7 (13%)
(iii) Ontario	31 (56%)
(iv) Western Provinces (MB, AB, BC)	12 (21%)

Specialty:	
(i) Gastroenterology	43 (78%)
(ii) Not specified/other	11 (20%)
(iii) General surgery	1 (2%)

Practice setting:	
(i) Academic	32 (58%)
(ii) Community	23 (42%)

Nonclinical roles:^*∗*^	
(i) Education	32 (58%)
(ii) Research	21 (39%)
(iii) Administrative	11 (20%)

Patients seen monthly with chronic constipation	
(i) <10	12 (22%)
(ii) 11–25	29 (53%)
(iii) 26–50	13 (23%)
(iv) 51–75	0 (0%)
(v) 76–100	0 (0%)
(vi) >100	1 (2%)

Majority of respondents were from Ontario and practiced in gastroenterology. More than half of the respondents saw at least 11 patients with chronic constipation on a monthly basis. ^*∗*^Respondents could select more than one response.

**Table 2:** Current recommended dosages of pharmacological agents for the treatment of constipation.

Type	Agent	Dose
Prosecretory agent	Linaclotide	290 *μ*g oral daily (IBS-C); 145 *μ*g oral daily (CIC)

Fibre supplement	Psyllium	12 g daily or as tolerated; incremental increases of 3-4 g/week to achieve target consumption

Prokinetic agent	Prucalopride	2 mg oral daily; dose reduces to 1 mg oral daily in patients ≥65 years of age or renal insufficiency with creatinine clearance ≤30 mL/min

Osmotic laxative	Lactulose	15–30 mL oral twice daily as needed
	PEG 3350	17 g/day as needed
	Magnesium hydroxide^*∗*^	15–30 mL (80 mg/mL) oral daily as needed

Stimulant laxative	Sodium picosulfate	10 mg once daily
	Senna	8.6–17.2 mg once daily
	Bisacodyl	5–15 mg once daily

^*∗*^Avoid in patients with chronic renal insufficiency.

## Appendix

### Chronic Constipation Questionnaire

How would you describe your current work environment?
  □ Academic
  □ Community
  □ Other: —
What is your current speciality?
  □ General Surgery
  □ GI specialist
  □ Internal medicine
  □ Family Practitioner
  □ Other: —
Please identify any other role you have (select all that apply)
  □ Educator
  □ Researcher
  □ Administrator
  □ Other: —
(1) How many patients with chronic constipation do you see on a monthly basis?
  □ None
  □ <10
  □ 11–25
  □ 26–50
  □ 51–75
  □ 76–100
  □ 100+
(2) Do you generally follow a standard, graduated or stepwise approach to the management of all patients with constipation?
  □ Yes
  □ No
(3) In your experience, do you think that referring physicians follow a standard, graduated or stepwise approach to the management of patients with constipation?
  □ Yes
  □ No
(4) Based on the type of chronic constipation, please mark the order in which the patient would ideally receive the following categories or treatment?* Please order from 1–6 (1 = first choice, 6 = last choice)*
  Inadequate fibre intake
    Stimulant laxatives …
    Stool softeners …
    Osmotic laxatives …
    Prokinetic (5-HT4 agonist) …
    Fibre supplement/bulking agent …
    Pro-secretory Agent (GCC agonist) …
    Other agents …
  CIC, Slow Transit
    Stimulant laxatives …
    Stool softeners …
    Osmotic laxatives …
    Prokinetic (5-HT4 agonist) …
    Fibre supplement/bulking agent …
    Pro-secretory Agent (GCC agonist) …
    Other agents …
  CIC, Functional Outlet Obstruction (ie. Pelvic floor dysfunction)
    Stimulant laxatives …
    Stool softeners …
    Osmotic laxatives …
    Prokinetic (5-HT4 agonist) …
    Fibre supplement/bulking agent …
    Pro-secretory Agent (GCC agonist) …
    Other agents …
  IBS-C
    Stimulant laxatives …
    Stool softeners …
    Osmotic laxatives …
    Prokinetic (5-HT4 agonist) …
    Fibre supplement/bulking agent …
    Pro-secretory Agent (GCC agonist) …
    Other agents …
(5) Do you feel there is reluctance to move from over-the-counter therapies to prescription agents from:
  (A) Patients
    □ Yes
    □ No
  (B) Referring Physicians
    □ Yes
    □ No
(6) How satisfied are you with the following treatment options when managing patients with (A) CIC and (B) IBS-C? (1 being not satisfied, 3 being neutral, 5 being very satisfied)
  Stimulant Laxatives
    □ 1
    □ 2
    □ 3
    □ 4
    □ 5
  Stool Softeners
    □ 1
    □ 2
    □ 3
    □ 4
    □ 5
  Osmotic Laxatives
    □ 1
    □ 2
    □ 3
    □ 4
    □ 5
  Prokinetic (5-HT4 agonist)
    □ 1
    □ 2
    □ 3
    □ 4
    □ 5
  Fibre supplement/bulking agent
    □ 1
    □ 2
    □ 3
    □ 4
    □ 5
  Pro-secretory Agent (GCC agonist)
    □ 1
    □ 2
    □ 3
    □ 4
    □ 5
(7) Based on the type of constipation, please identify which symptoms you feel most affect your patients quality of life? (Order symptoms from 1–5, 1 being the most burdensome, 5 being the least)
  CIC
    Abdominal pain/bloating …
    Straining during bowel movements …
    Sensation of incomplete evacuation …
    Sensation of anorectal obstruction/blockage …
    Infrequent stools …
  IBS-C
    Abdominal pain/bloating …
    Straining during bowel movements …
    Sensation of incomplete evacuation …
    Sensation of anorectal obstruction/blockage …
    Infrequent stools …
(8) In your experience, which symptoms (if any) do patients most often complain about while on the following therapy options? (Please select all that apply)
  Stimulant Laxatives
    Abdominal pain/bloating …
    Straining during bowel movements …
    Sensation of incomplete evacuation …
    Sensation of anorectal obstruction/blockage …
    Infrequent stools …
  Stool Softeners
    Abdominal pain/bloating …
    Straining during bowel movements …
    Sensation of incomplete evacuation …
    Sensation of anorectal obstruction/blockage …
    Infrequent stools …
  Osmotic Laxatives
    Abdominal pain/bloating …
    Straining during bowel movements …
    Sensation of incomplete evacuation …
    Sensation of anorectal obstruction/blockage …
    Infrequent stools …
  Prokinetic (5-HT4 agonist)
    Abdominal pain/bloating …
    Straining during bowel movements …
    Sensation of incomplete evacuation …
    Sensation of anorectal obstruction/blockage …
    Infrequent stools …
  Fibre supplement/bulking agent
    Abdominal pain/bloating …
    Straining during bowel movements …
    Sensation of incomplete evacuation …
    Sensation of anorectal obstruction/blockage …
    Infrequent stools …
  Pro-secretory Agent (GCC agonist)
    Abdominal pain/bloating …
    Straining during bowel movements …
    Sensation of incomplete evacuation …
    Sensation of anorectal obstruction/blockage …
    Infrequent stools …
(9) Are you aware of the new ACG 2014 recommendations on the management of IBS-C and Chronic-Idiopathic Constipation from 2014?
  □ Yes
  □ No
  (a) If yes, how useful are the ACG guidelines in helping you prioritize how available agents are used? (1 being not useful, 3 being neutral, and 5 being very useful)
    □ 1
    □ 2
    □ 3
    □ 4
    □ 5
  (b) How valuable do you feel an evidence based treatment algorithm that translates the ACG guidelines into Canadian clinical practice would be? (1 being not valuable, 3 being neutral, 5 being very valuable)
    □ 1
    □ 2
    □ 3
    □ 4
    □ 5
